# Intralabyrinthine MRI FLAIR as a predictive marker for hearing loss in vestibular schwannomas in Neurofibromatosis Type 2

**DOI:** 10.1007/s11060-026-05422-9

**Published:** 2026-01-26

**Authors:** Robert L. Walker, Maxwell T. Laws, H. Jeffrey Kim, Christopher Zalewski, Ashok Asthagiri, Sruthi Ranganathan, Christina Hayes, John D. Heiss, John A. Butman, Prashant Chittiboina

**Affiliations:** 1 Neurosurgery Unit for Pituitary and Inheritable Disorders, National Institute for Neurological Diseases and Stroke, Bethesda, MD USA; 2 Department of Radiation Oncology, Thomas Jefferson University, Philadelphia, PA USA; 3 Surgical Neurology Branch, National Institute of Neurological Disorders and Stroke, Bethesda, MD USA; 4 Department of Neurosurgery, University of Virginia, Charlottesville, VA USA; 5 National Institute on Deafness and Other Communication Disorders, Bethesda, MD USA; 6 Department of Otolaryngology-Head and Neck Surgery, Georgetown University Hospital, Washington, DC USA; 7 Audiology Branch, Otolaryngology Branch, National Institute on Deafness and Other Communication Disorders, Bethesda, MD USA; 8 Department of Medicine, University of Cambridge, Cambridge, UK; 9 Radiology and Imaging Sciences, Clinical Center at the National Institutes of Health, Bethesda, MD USA

**Keywords:** Acoustic neuroma, Hearing loss, Labyrinthine protein, MRI FLAIR, NF2, Neurofibromatosis 2, Vestibular schwannoma, Perilymph, Tumor growth

## Abstract

**Background and objectives:**

The onset of hearing loss due to vestibular schwannomas (VS) is inevitable but does not correlate with the size of the tumor. In patients with Neurofibromatosis Type 2 (NF2) and VS, we previously found an association between pre-contrast fluid-attenuated inversion recovery magnetic resonance imaging (FLAIR MRI) signal in the labyrinth and hearing loss. Here, we asked whether FLAIR hyper-intensity could serve as a predictive biomarker for hearing loss in NF2 patients with VS.

**Methods:**

A prospective longitudinal study (NCT00598351) of NF2 enrolled 168 subjects between 2008 and 2013. This study included 34 patients with small VS (total volume ≤ 500mm^3^). Middle fossa decompression surgery (*n* = 4 patients) was provided via a standard-of-care trial (NCT00060541).

**Results:**

From 34 eligible subjects (mean age 26.8y) with NF2 and small VS, 53 ears met inclusion criteria. Abnormal hearing was recorded in 18 ears at study entry; all 18 ears had FLAIR hyper-intensity. Of the 35 ears with normal hearing, 16 had FLAIR hyper-intensity at study entry, 6 (37.5%) of which developed new hearing loss (median time to hearing loss of 4.45 years). Conversion to FLAIR hyper-intensity occurred in 11 ears, 3 of which proceeded to hearing loss. No hearing loss developed in the eight ears that remained FLAIR negative. FLAIR conversion has high sensitivity (1·00, 95% CI 0·39–1) and negative predictive value (1·00, 95% CI 0·63–1) for new-onset hearing loss. In 4 patients undergoing middle fossa decompression surgery, we found that surgery stabilized hearing but did not reverse FLAIR hyperintensity.

**Conclusions:**

Our findings suggest that intralabyrinthine FLAIR hyper-intensity is a sensitive, non-invasive biomarker for hearing loss related to VS. Hearing decline followed FLAIR hyper-intensity by approximately 4 years but was absent in ears with normal FLAIR signal.

**Trial registration:**

Registry: ClinicalTrials.gov, TRN: NCT00060541, Registration date: 4 June 2003.

**Supplementary Information:**

The online version contains supplementary material available at 10.1007/s11060-026-05422-9.

## Introduction

Predicting the onset of hearing loss in patients with vestibular schwannoma (VS), either sporadic or in the context of neurofibromatosis type 2 (NF2) is a challenging clinical problem [1, 2]. Longitudinal analysis in NF2 related VS (NF2-VS) showed that hearing loss correlates weakly with tumor size and growth rate [1, 3, 4]. The multilobulated, polyclonal VSs of NF2 patients have unpredictable growth patterns [5, 6]. Although, VS results in near universal hearing loss in NF2 [7, 8], the exact mechanisms remain poorly understood [9]. Cochlear aperture obstruction and elevated intralabyrinthine protein has been proposed as a unifying mechanism for hearing loss from VS of various sizes [9].

Historically, high protein content within surgically sampled perilymph has been associated with hearing loss due to VS [10, 11]. More recently, non-invasive techniques, including fluid-attenuated inversion recovery (FLAIR) magnetic resonance imaging (MRI), have been used to detect elevated protein levels within in-vivo fluid compartments, including cerebrospinal fluid [12]. Our prior study involving NF2 patients found an association between intralabyrinthine FLAIR hyper-intensity (corresponding to elevated protein) and hearing loss [9]. Interestingly, while no patients with normal FLAIR labyrinthine signal experienced hearing loss (high negative predictive value), some patients with intralabyrinthine FLAIR hyper-intensity still maintained normal hearing. These observations implied that FLAIR hyper-intensity could precede, and possibly predict, the hearing loss in NF2-VS.

This study aimed to prospectively evaluate the predictive power of FLAIR MRI for hearing loss in NF2 patients with small, treatment-naïve VS.

## Methods

### Patient population

Patients with NF2 were enrolled in a prospective, longitudinal, natural history study at the National Institutes of Health (NIH) Clinical Center (CC) in Bethesda, MD, between 2008 and 2013 with a 5-year follow-up. The Combined Neuroscience Institutional Review Board of the NIH CC approved the clinical research protocol. All participants gave informed consent to participate in the study. Patients underwent yearly MRI studies, comprehensive audiometric assessments, and clinical follow-up at the NIH CC.

### Inclusion criteria

NF2 patients with at least one untreated VS with a total tumor volume (i.e., internal auditory canal (IAC) and posterior fossa (PF) components) of ≤ 500mm^3^ and more than one follow-up evaluation were included. We limited inclusion to small VS to minimize confounding from surgery or large-tumor effects on the labyrinthine FLAIR signal. Exclusion criteria were bilateral surgery or radiosurgery for VS, other diagnoses resulting in hearing loss, and initial total tumor volume > 500mm^3^. Four patients in this study underwent middle fossa decompression (MFD) for hearing preservation [13–15] as per standard-of-care in another trial (NCT00060541).

### Imaging evaluation

Patients underwent MR imaging with and without gadolinium contrast of the brain and spine. Pre-contrast FLAIR was obtained as described before [9]. Posterior fossa was imaged with an in-plane resolution of less than 1 mm with a 1.5 Tesla MR scanner (Phillips, Andover, MA). VS tumor component measurements (i.e., IAC and PF) were performed on volumetric post-contrast MR T1-weighted images. VS tumor component volume was calculated using the ellipsoid equation: $\:V=\:\frac{A\:x\:B\:x\:C}{2}$ where A, B, and C were the maximum length measured in the medial-lateral, anterior-posterior, and cranial-caudal directions [16]. Total VS tumor volume was obtained by adding the volumes of IAC and PF components, using the ridge of the petrous bone as the dividing line [3, 9]. IAC occlusion was noted if there was an absence of fluid signal around the VS within the IAC on high-resolution T2-weighted and BFFE images. Specific growth rate (SGR) was used to analyze the VS growth rate [17, 18]. $\:SGR\:=\:\frac{\mathrm{ln\:}(V\mathrm{2}/V\mathrm{1})}{(t\mathrm{2}-t\mathrm{1})}\:$ where *V* is tumor volume measured at two time (t) points. SGR is the growth constant of the tumor and has the units of %/day. SGR was also calculated separately for IAC, PF, and total volume tumor components.

FLAIR MRI sequences were performed for each patient during every follow-up visit to assess for elevated labyrinthine protein [9]. All studies used a standardized IAC FLAIR protocol, consisting of an inversion-recovery–based, multi-shot turbo spin echo acquisition (TR 11,000 ms; TE 120 ms; inversion delay 2,550 ms; TSE factor 26; voxel size 0.47 × 0.64 × 1.80 mm; field of view 320 × 234; slice thickness 1.8 mm; SPIR fat suppression; NSA 4). Pre-contrast FLAIR images were analyzed by a board-certified neuroradiologist (JB) blinded to the clinical outcome data. Labyrinthine FLAIR hyperintensity was reported as “positive” when inner-ear signal exceeded adjacent brain parenchyma and “negative” when signal intensities were comparable.

### Hearing loss

Detailed history and physical exam were obtained at each protocol-related neurosurgical clinic visit. Each visit also included evaluation by a board-certified neuro-otologist (HJK) and comprehensive audiometric and vestibular testing by nationally certified audiologists (CZ, CB).^3^ Audiometric evaluations measured air conduction (AC) thresholds from 250 to 8000 Hz and bone conduction (BC) thresholds from 250 to 4000 Hz. Hearing loss was defined using the modified World Health Organization [19] four-frequency pure tone average (4f-PTA) of 0.5, 1, 2, 4 kHz classification as follows: Normal hearing (≤ 20dB HL), Mild loss (> 20 and ≤ 40dB HL), Moderate loss (> 40 and ≤ 70dB HL), Severe loss (> 70 and ≤ 95dB HL), Profound loss (> 95dB HL) [12, 20]. Hearing decline was defined as a decline of AC/BC 4f-PTA by 10dB from baseline during the study. Baseline and later follow-up audiometric evaluations were compared to determine if hearing decline occurred.

### MFD cohort

For patients undergoing MFD surgery, hearing outcomes, 4f-PTA and word recognition score (WRS) from pre-operative audiogram, obtained within a month prior to surgery were compared to the post-operative outcomes obtained at one year follow-up. Average follow-up was 1.9 ± 1.3 years. Intralabyrinthine FLAIR hyper-intensity was assessed on all pre-operative and post-operative MRI studies. Three of the four patients who underwent MFD had both pre-operative (within seven days prior to surgery) and post-operative (one year follow-up) MRI studies obtained. These MRIs were analyzed for labyrinthine FLAIR signal hyper-intensity. Time-to-event analyses for hearing decline were performed using Kaplan–Meier survival methods, with comparisons between groups assessed using the log-rank test. Survival curves were generated, and median survival times were reported when estimable.

### Statistical analysis

Ears were analyzed independently. Continuous variables (age, tumor volume, and specific growth rate) were summarized as mean ± standard deviation and compared using unpaired t-tests after assessment of normality with the Shapiro–Wilk test; equality of variance was verified where applicable. Categorical variables (IAC occlusion, intralabyrinthine FLAIR status, hearing category) were analyzed using Chi-square or Fisher’s exact tests, depending on expected cell counts. Sensitivity, specificity, and predictive values were calculated with corresponding 95% confidence intervals.

Time-to-event analyses for hearing decline were performed using Kaplan–Meier survival estimation, stratified by baseline intralabyrinthine FLAIR status. Groups were compared using the two-sided log-rank test, and median survival times were reported when estimable. All survival analyses were conducted in R using the survival and survminer packages. All statistical tests were two-tailed with significance defined as *p* < 0.05.

### Data availability statement

The human data generated in this study are not publicly available due to patient privacy requirements but are available upon reasonable request from the corresponding author. Other data generated in this study are available within the article and its Supplemental data files.

## Results

### Patient demographics

**Fig. 1:**
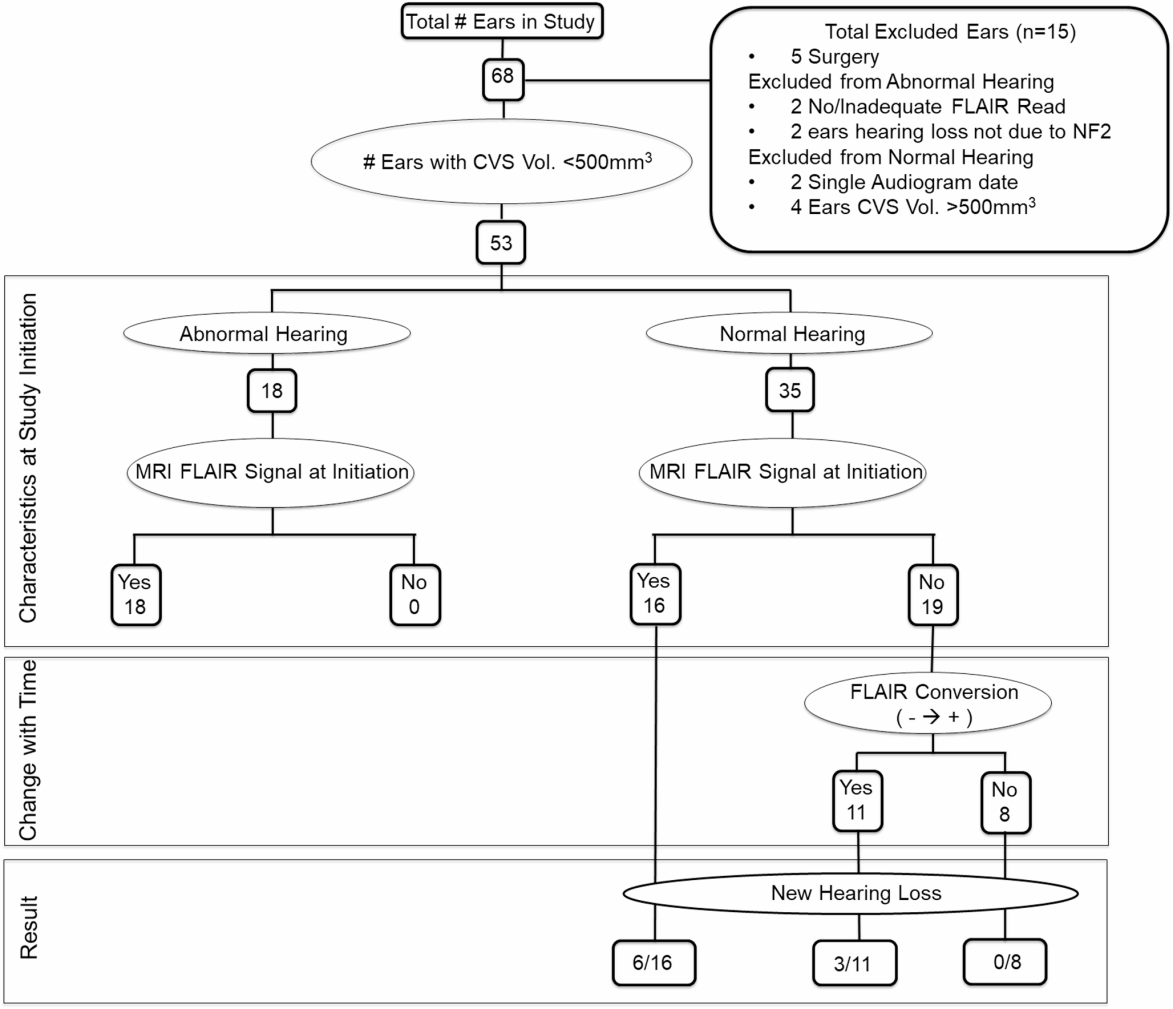
Hearing loss and imaging findings in NF2. This flow diagram is a graphical representation of the current study. The ears excluded from the study are first highlighted. At baseline, the ears were dichotomized according to hearing status. Intralabyrinthine FLAIR hyper-intensity was then evaluated. The ears were then followed in time for both changes in hearing, IAC occlusion, and intralabyrinthine FLAIR signal intensity. The flow diagram is represented as three key zones during the study: characteristics at study initiation (top box), changes during the study (middle box), and the hearing status at study end (bottom box). A change in intralabyrinthine FLAIR from normal to hyperintense (FLAIR Conversion) is designated as - → +

From the 168 subjects enrolled, 34 with small VS tumors were included in this study. The rest had large VS tumors, prior surgeries, or profound hearing loss, and consequently were excluded from this study. The 34 study participants ranged from 4.6 to 67 years, with an average age of 26.8 years at study onset. Of the study population, nine were males and 25 were females. Mean follow-up for all participants was 4.2 ± 1.2 years, with an average of 6.3 visits for imaging, audiometric, and clinical evaluations. We excluded 15 out of the 68 ears for the following reasons: five due to previous surgeries, two for inadequate FLAIR images, two for hearing loss unrelated to NF2, two with only one evaluable audiogram, and four due to a VS volume exceeding 500mm^3^ (Fig. 1). Neither the patient’s age (*p* = 0.93) nor gender (*p* = 0.46) correlated with hearing loss at the study onset (Table 1).

**Table 1:** Summary of the study subjects and their characteristics at study entry

	Normal Hearing (*n* = 26)	Hearing Loss (*n* = 9)	*p*-value	95% Confidence Interval
**Age**				
Baseline Age (years)	21.04 *±* 13.77	21.56 *±* 16.43	0.93	-10.86 to 11.89
**Sex**				
Males	13	3	0.46	
Females	13	6		
**Volume (mm** ^**3**^ **)**				
*Internal Auditory Canal (IAC)*				
Initial	103.5 *±* 113.5 (*n* = 26)	146.9 *±* 110.7 (*n* = 9)	0.33	-45.34 to 132.2
End	190.4 *±* 161.7 (*n* = 26)	349.6 *±* 223.3 (*n* = 9)	0.03*	17.86 to 300.3
Volume Change	86.96 *±* 100.6 (*n* = 26)	202.7 *±* 151.9 (*n* = 9)	0.01*	25.06 to 206.3
*Posterior Fossa (PF)*				
Initial	40.9 *±* 108.4 (*n* = 26)	146.7 *±* 323.9 (*n* = 9)	0.15	-40.1 to 251.5
End	636.6 *±* 1454.1 (*n* = 26)	1884.5 *±* 4800.2 (*n* = 9)	0.24	-861.6 to 3357
Volume Change	595.6 *±* 1402.1 (*n* = 26)	1738 *±* 4487.8 (*n* = 9)	0.25	-844 to 3128
*Total Tumor (IAC + PF)*				
Initial	144.5 *±* 196.4 (*n* = 26)	293.6 *±* 429.4 (*n* = 9)	0.16	-64.8 to 363.1
End	827.1 *±* 1580.7 (*n* = 26)	2234.1 *±* 4992.6 (*n* = 9)	0.21	-809.5 to 3624
Volume Change	682.5 *±* 1442.5 (*n* = 26)	1940 *±* 4582.8 (*n* = 9)	0.22	-773.9 to 3290
**Specific Growth Rate (SGR**,** %/day)**				
IAC	0.00045 *±* 0.00078 (*n* = 26)	1.15 *±* 3.45 (*n* = 9)	0.09	-0.1862 to 2.486
PF	0.0018 *±* 0.0013 (*n* = 7)	0.0018 *±* 0.0011 (*n* = 4)	0.99	-0.0018 to 0.0018
Total	0.00076 *±* 0.00092 (*n* = 26)	0.0011 *±* 0.00072 (*n* = 9)	0.33	-0.0003562 to 0.001023
**IAC Occlusion**				
Baseline Normal Hearing Ears	73% (*n* = 19)	100% (*n* = 9)	0.08	

• * Indicates significance *p* < 0.05

• Unpaired t-test for Volume, SGR, and Age analysis

• PF SGR has smaller n because SGR could not be calculated for ears having initial volume (V_i_) of 0

• Chi-Square used for the IAC Occlusion vs. Hearing Status

### Effect of component VS volume and growth rate on hearing loss

We evaluated changes in VS volume by analyzing total, IAC, and PF components. For the total VS, we found no significant difference in the mean initial volume, final volume, and volume change among participants with or without hearing decline (Table 1). This outcome affirms that total VS tumor volume is an unreliable hearing predictor, as noted previously by Massick et al. [11]. Additionally, the PF component showed no association with hearing loss. Similarly, there was no association between initial IAC component volume and hearing loss (*p* = 0.33). However, we discovered an association between hearing decline, and both the final IAC volume (*p* = 0.03) as well as a change in IAC volume (*p* = 0.01) (Table 1). These results suggest a link between the growth of the IAC component and hearing decline. To determine if hearing decline was exclusively due to biological factors (e.g., secreted proteins) common in faster-growing tumors, we conducted a quantitative analysis using the logarithmic specific growth rate (SGR) model [17, 18]. Regression analysis showed that the SGR at the start of the study was significantly lower in older patients (*p* = 0.04, Supplemental Figure S1A). There were no significant differences in SGR between participants without (*n* = 26) and with hearing decline (*n* = 9) in the associated ears (*p* = 0.95, Supplemental Figure S1B). Consistent with our earlier work [19], these results indicate that while IAC tumor volume correlates with hearing loss, higher VS growth rates alone were not predictive of hearing decline.

### IAC occlusion underlies intralabyrinthine FLAIR hyper-intensity and hearing decline

In an earlier cross-sectional analysis of the current cohort, we have shown that the IAC component of VS may lead to the occlusion of the IAC. Occlusion of the IAC was associated with both intralabyrinthine FLAIR hyper-intensity and hearing loss [9]. In the current study, we found that IAC occlusion at study entry was associated with intralabyrinthine FLAIR hyper-intensity (Fisher’s exact test; *P* < 0.0001, RR CI 2.0 - ∞, Supplemental Figure S2) but not with hearing decline. All ears with hearing loss at study initiation had IAC occlusions and intralabyrinthine FLAIR hyper-intensity (18/18 ears). All ears with new FLAIR hyper-intensity had IAC occlusion by the study end (27/27). IAC occlusion was highly specific (0.88, 95% CI 0.53–0.99) and positively predictive (0.96, 95% CI 0.82–1) for the development of intralabyrinthine FLAIR hyper-intensity. IAC occlusion also had high sensitivity (1, 95% CI 0.70–1) and negative predictive value (1, 95% CI 0.65–1) for hearing decline. Taken together, these observations are consistent with a temporal sequence in which IAC occlusion precedes the development of intralabyrinthine FLAIR hyper-intensity and the later onset of hearing decline.

### Intralabyrinthine FLAIR hyper-intensity precedes hearing decline

**Fig. 2:**
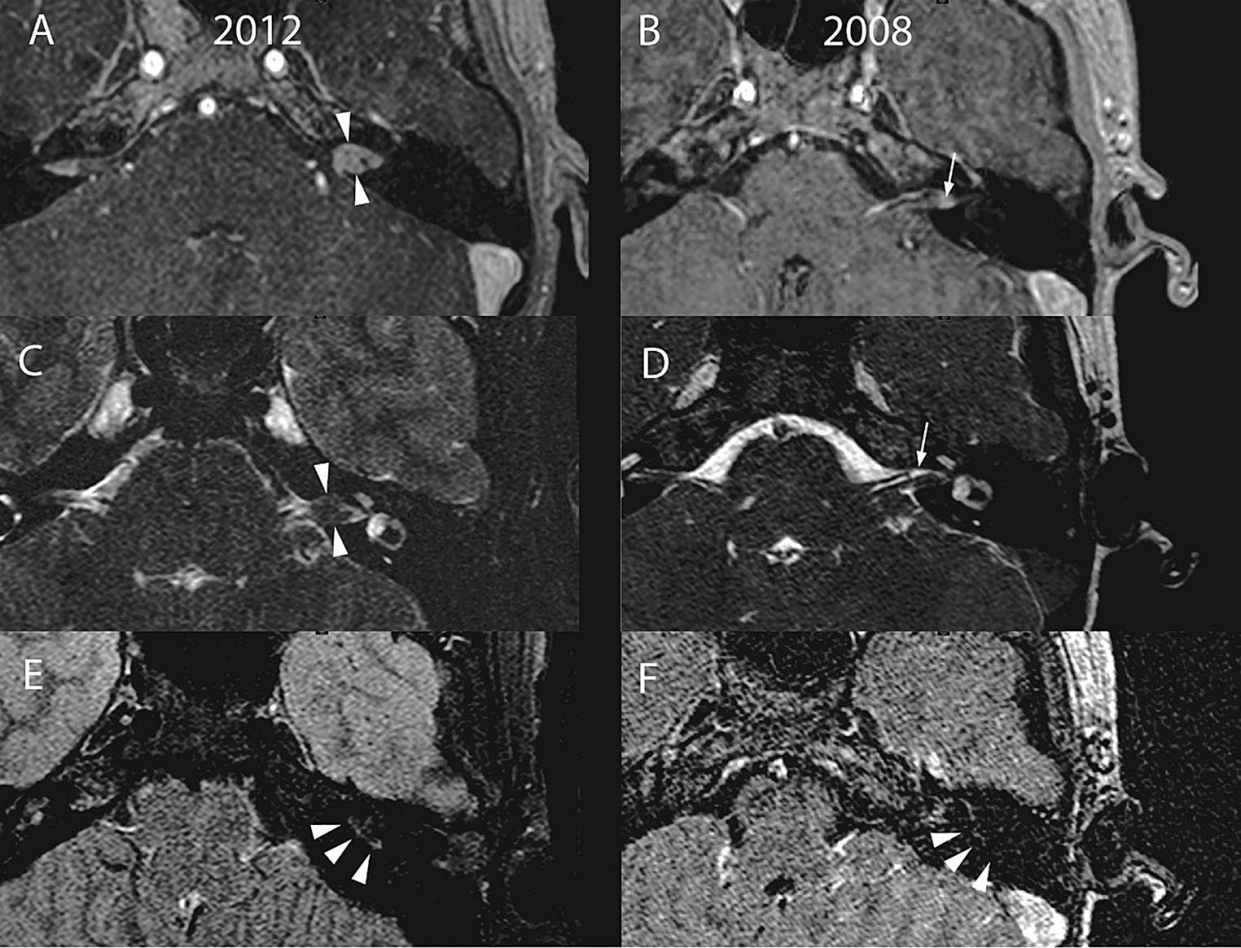
Imaging of vestibular schwannomas in the study. Illustrative MRI images from a single patient at study initiation (y:2008) and at study termination (y:2012). **A** and **B.** Over the study period, T1-weighted post-contrast images show the growth of a small, cochlea-vestibular schwannoma (white arrow in panel B) within the left internal auditory canal grew to encompass the entire canal (white arrowheads in panel A). **C** and **D.** T2-weighted imaging through the same axial sections reveal occlusion of the internal auditory canal (white arrowheads in C) with tumor, but not in D (white arrow). **E** and **F.** Pre-contrast FLAIR images from an axial section 3 mm below the canal reveal appearance of FLAIR hyper-intensity in the basal turn of the cochlea (white arrowheads in E). Expected FLAIR appearance of basal turn of cochlea in a normal ear (white arrowheads in F)

We wanted to study whether intralabyrinthine FLAIR hyper-intensity preceded hearing decline (Fig. 2, Supplemental Figure S3). Such a finding could establish the prognostic value of non-invasive MRI monitoring of intralabyrinthine FLAIR signal in predicting hearing loss. First, we observed that all 18 ears presenting with hearing loss at study entry also demonstrated intralabyrinthine FLAIR hyper-intensity (18/18, 100%). We also found that in the ears with baseline normal hearing (n = 35), some ears (16/35, 46%) demonstrated intralabyrinthine FLAIR hyper-intensity and the rest (19/35, 54%) had a normal intralabyrinthine FLAIR signal. Of these 19 FLAIR ‘normal’ ears, 11 converted to a hyper-intense FLAIR signal, and eight remained FLAIR ‘normal’ by the study end (Fig. 1).

None of the ears (0/8) that remained FLAIR ‘normal’ during the study developed hearing loss. Baseline intralabyrinthine FLAIR hyper-intensity was associated with hearing decline in 37.5% (6/16). A smaller proportion (3/11, 27.2%) of ears with FLAIR conversion developed hearing decline during the study. FLAIR conversion showed high sensitivity (1.00, 95% CI 0.39–1) and negative predictive value (1.00, 95% CI 0.63–1) for hearing decline. However, FLAIR conversion was not specific (0.47, 95% CI 0.22–0.72) and had a low positive predictive value (0.30, 95% CI 0.09–0.61) for hearing loss. Lastly, once FLAIR hyper-intensity developed, it persisted in all cases. These findings suggest that intralabyrinthine FLAIR hyper-intensity was a non-reversible event associated with later hearing decline.

### Time to hearing decline

We found that IAC occlusion was associated with intralabyrinthine FLAIR hyper-intensity and hearing loss. We next examined the temporal relationship of these factors. FLAIR conversion occurred in 11 ears during the study (1.7 ± 1.7 years). Of these 11 ears, three developed hearing loss within 2.23 ± 1.54 years from FLAIR conversion.

**Fig. 3:**
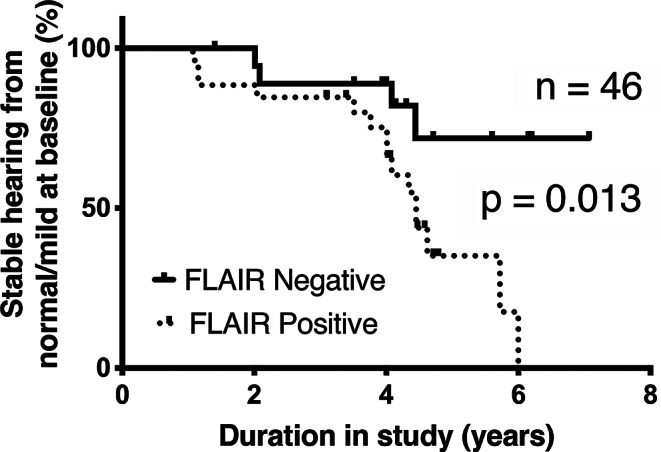
Hearing loss occurs with intralabyrinthine FLAIR hyper-intensity. Kaplan Meier analysis of hearing decline reveals that ears that converted from ‘normal’ intralabyrinthine FLAIR signal developed hearing loss during the study. The Logrank (Mantel-Cox) test was significantly different for the tested groups (Chi-Square 6.11, *p* = 0.01)

A Kaplan Meier analysis (Fig. 3) confirmed that hearing decline occurred sooner (median survival of normal hearing − 4.45 years into the study) in patients with baseline FLAIR hyper-intensity at study entry (*p* = 0.01). In ears with baseline normal FLAIR, hearing loss always followed FLAIR conversion, but median survival was not reached. These findings suggest that intralabyrinthine FLAIR hyper-intensity may precede the onset of hearing decline.

### MFD does not reverse intralabyrinthine FLAIR hyper-intensity

NF2 patients with VS tumors and intralabyrinthine FLAIR hyper-intensity (*n* = 4) underwent MFD to postpone hearing loss. There were no significant changes found in the rate of hearing decline as measured by 4f-PTA and word recognition percentage before and after surgery (Supplemental Fig. 4, 4f-PTA before: +0.729 dB/year v. after: +4.78 dB/year, *p* = 0.52; word recognition percentage before: 5.76%/year v. after: -0.75%/year, *p* = 0.11). This is further supported by a linear regression analysis on both scores showing no significant differences in the rate of hearing decline changes after surgery by frequency (*p* = 0.375; *p* = 0.614). FLAIR MRI sequences were analyzed at one year following surgery. Surgical intervention did not reverse intralabyrinthine FLAIR hyper-intensity in any of the ears undergoing the intervention (Fig. 5).

### Discussion

Vestibular schwannomas and hearing loss are a significant source of morbidity in NF2 [21]. Hearing loss occurs in 95% of patients with NF2 and is related to the presence of VS [22]. However, VS tumor size and growth rate remain poor predictors of hearing loss in NF2-VS [7, 10, 11]. Early detection and prediction of hearing loss in these patients is critical to developing therapies that may delay or prevent these clinical sequelae.

### Relevance of VS size and growth in hearing loss

**Fig. 4:**
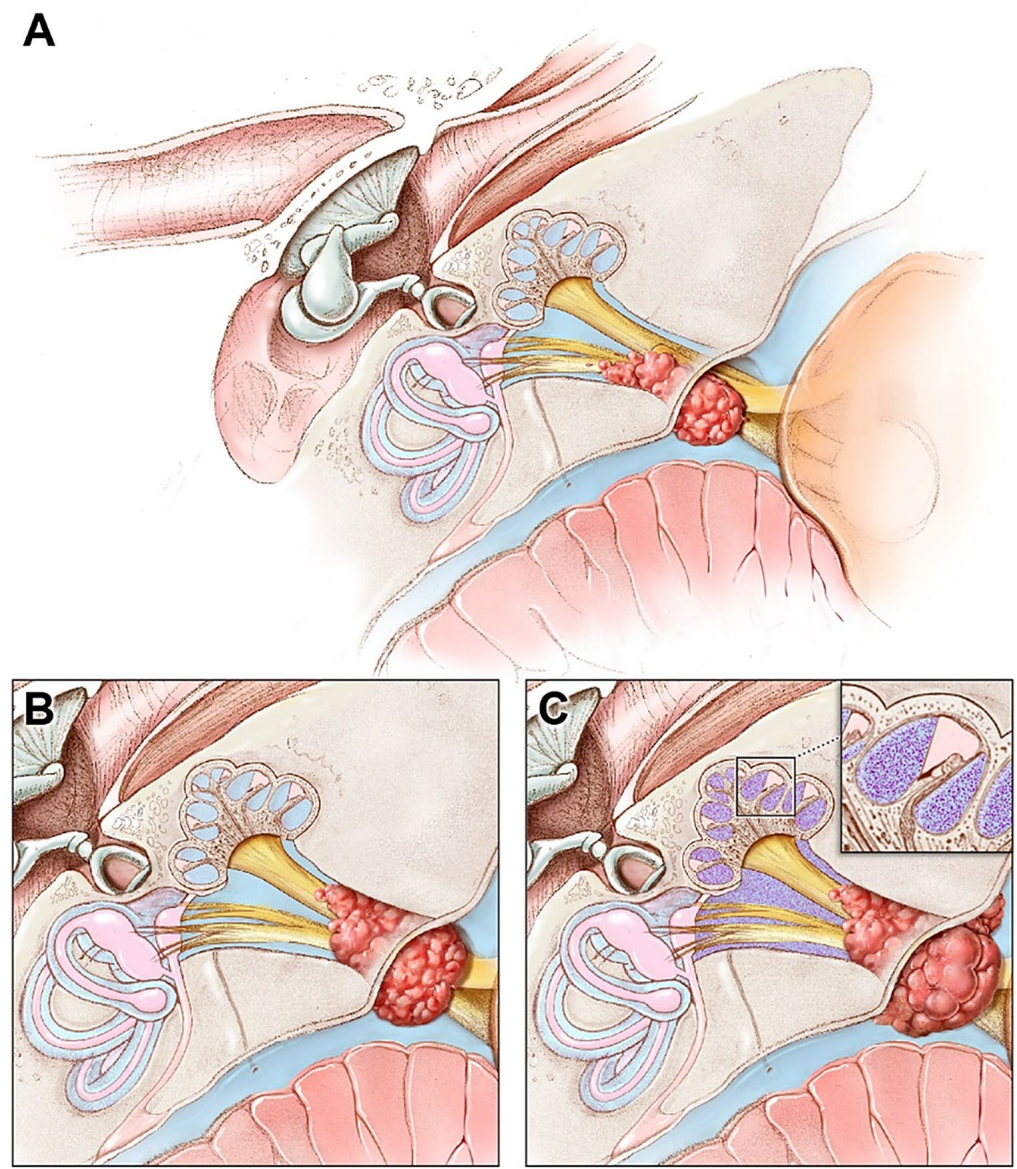
Mechanism of hearing loss in NF2. **A**. The VS grows by coalescing multiple tumorlets, eventually occluding the IAC. **B**. A key intermediary event is IAC occlusion. **C**. Following IAC occlusion, intralabyrinthine protein accumulates within the perilymph due to reduced outflow, which we suspect contributes to hearing loss. Intralabyrinthine protein accumulation is seen as FLAIR abnormality (C inset)

Our findings confirm hearing loss is independent of VS size [12] and that tumor growth declines with age [20]. In this study of small, treatment-naïve VS tumors, the final IAC component volume of VS tumors emerged a critical factor associated with hearing changes. This builds on our previous work where we found a relationship between IAC occlusion by the tumor, intralabyrinthine FLAIR hyper-intensity, and hearing loss (Fig. 4) [12]. We suspected that the growth of the IAC component led to occlusion of the IAC and possibly raised intracanalicular pressure [21], Raised intracanalicular pressure could be the precursor for intralabyrinthine protein accumulation and eventual hearing loss [10, 11, 22]. However, measuring intracanalicular pressure and obtaining perilymph for protein content analysis are highly invasive procedures with inherent risks of hearing loss [11, 21].

### Intralabyrinthine FLAIR hyper-intensity as a predictive biomarker

We explored the use of intralabyrinthine FLAIR hyper-intensity as a predictive biomarker for NF2-related hearing loss via the detection of intralabyrinthine protein accumulation. Our findings suggest ears that do not develop intralabyrinthine FLAIR hyper-intensity are not at risk for hearing loss. Ears that had detectable intralabyrinthine FLAIR hyper-intensity were at risk for developing hearing loss. Of the 16 ears at risk, six developed hearing loss during the study. During the investigation, some ears with FLAIR conversion also developed hearing loss (3/11). We observed an approximate 2-year risk of developing hearing loss from the time of FLAIR hyper-intensity detection. Ears with baseline hyper-intensity took a median of 4.45 years to develop hearing loss. Despite the limitations due to the low prevalence of NF2, we found a high probability of a FLAIR-positive signal when abnormal hearing was detected. Our study suggests that monitoring intralabyrinthine FLAIR hyper-intensity could aid hearing loss management strategies in NF2 patients. If this hyper-intensity appears on surveillance MRI imaging, therapies like bevacizumab or MFD could be started to prevent hearing loss [26–28].

A methodological limitation of this study is that FLAIR assessments were performed by a single board-certified neuroradiologist (JB) who pioneered FLAIR studies in patients with NF2. Although this blinded, single-reader design reduced interpretive bias and ensured consistency across longitudinal examinations, it does not allow assessment of inter-observer variability. Future studies incorporating multiple independent raters, and inter-rater agreement metrics, are essential to validate these findings across different readers, scanners, and institutions. While our standardized IAC FLAIR acquisition protocol enhanced reproducibility within the current cohort, more work is needed to determine how robust intralabyrinthine FLAIR measurements remain across imaging platforms. Together, these steps will be critical for confirming the generalizability of intralabyrinthine FLAIR hyperintensity as a biomarker of impending hearing decline.

### FLAIR hyper-intensity and protein accumulation

FLAIR hyper-intensity has been shown to rise with increasing cerebrospinal fluid (CSF) protein concentration. It implies that intralabyrinthine FLAIR hyper-intensity is likely indicative of protein accumulation within the perilymph [12]. Recent findings support assertion of a continuity of the CSF space with the cochlear perilymph [25]. Our findings suggest that the tumor-induced occlusion of the IAC could be a plausible mechanism leading to intralabyrinthine protein accumulation and FLAIR hyper-intensity on MRI (Fig. 4). Several hypotheses have been proposed to explain this protein accumulation, including fluid flow obstruction at the cochlear aperture, active protein secretion by the tumors, cochlear nerve compressive axonopathy, or arterial/venous occlusion at the IAC [22–29]. The temporal association between intralabyrinthine protein accumulation and hearing loss suggests that the pathophysiology of hearing loss depends on the VS disrupting inner ear homeostasis.

**Fig. 5:**
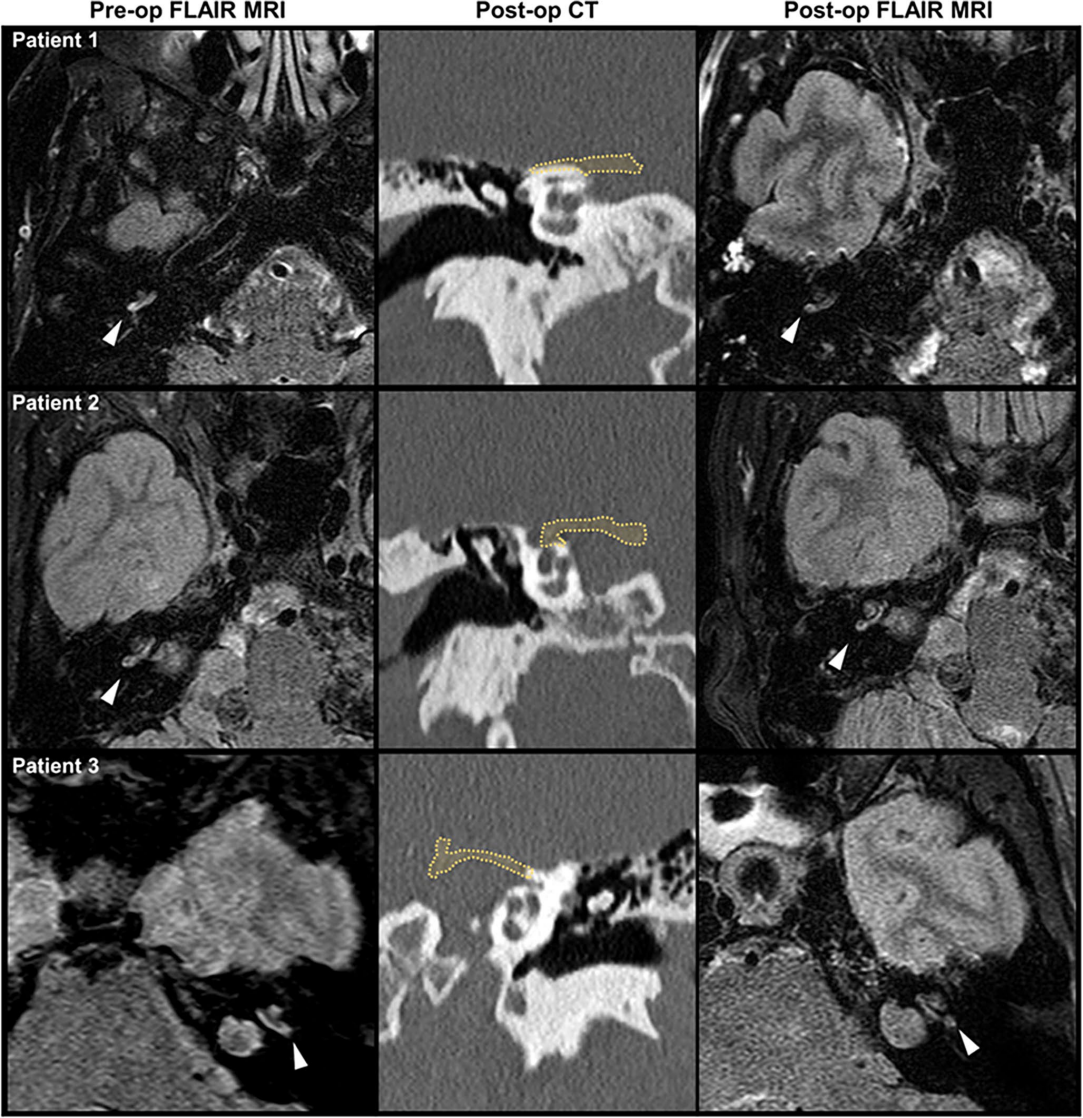
Middle fossa decompression does not reverse intralabyrinthine FLAIR hyper-intensity at one year follow-up. Pre-contrast axial FLAIR MRI images were obtained one to seven days prior to middle fossa decompression (left column) and one year following surgery (right column). Sections are through the basal turn of the cochlea (denoted by white arrowhead). Post-operative images show persistence of the FLAIR signal hyper-intensity within the basal segment of the cochlea in all three patients. The middle column represents post-operative coronal CT temporal bone through the cochlea and head of malleus. The yellow dashed line denotes the area of bone removed during the middle fossa decompression

Intralabyrinthine FLAIR hyper-intensity could serve as a predictive biomarker for eventual hearing loss in patients with VS, especially in the context of NF2. Currently, clinicians have a limited repertoire of interventions to recommend for hearing preservation, including bevacizumab or MFD surgery [13–15]. However, the optimal timing of such recommendation remains unexplored. The findings from this (Fig. 5) and prior studies [13–15] suggest that earlier interventions could help preserve hearing at a usable level. The observed stabilization of hearing following middle fossa decompression should be interpreted as an exploratory observation. Given the very limited number of patients (*n* = 4) and the absence of a control group, the study was not powered to determine treatment efficacy. These findings are descriptive and serve primarily to illustrate that decompression did not reverse intralabyrinthine FLAIR hyper-intensity, rather than to establish a causal or therapeutic effect. While these preliminary observations suggest that intervention at the time FLAIR hyper-intensity appears may merit further investigation, such hypotheses require rigorous evaluation in larger, controlled studies before clinical recommendations can be made. We posit that elevated intralabyrinthine protein could similarly predict imminent hearing decline in sporadic VS; however, dedicated studies in sporadic VS populations are required to establish such a mechanism in this population.

## Limitatons

The rarity of NF2 limits the ability to fully capture the natural course of FLAIR hyper-intensity from baseline to hearing loss development. As such, the findings may not be generalizable to a larger sporadic VS population. The pathogenesis of protein accumulation within the perilymph and FLAIR hyper-intensity remains unknown. While the tumor-induced occlusion of the IAC is a plausible mechanism, more research is required to confirm this and explore other potential factors involved. Lastly, while the study suggests the value of early intervention following a FLAIR change to preserve hearing, we did not observe a reversal of FLAIR signal with surgical intervention. This indicates that hearing may still worsen over time, even after intervention. It is therefore crucial to approach these interventions with caution and to monitor patients closely to evaluate their effectiveness.

## Conclusions

The findings from this study suggest that intralabyrinthine FLAIR hyper-intensity is a sensitive, non-invasive biomarker that precedes hearing loss in NF2 by approximately four years. Importantly, hearing loss is likely not to occur without FLAIR conversion in NF2. We find that occlusion of IAC likely leads to FLAIR hyper-intensity, and consequently hearing loss. The lack of reversal of FLAIR signal following surgical intervention highlights the potential inevitability of hearing decline, underscoring the importance of developing more effective strategies for hearing preservation. Future multicenter studies incorporating multi-reader reliability assessment and quantitative FLAIR signal analysis will be essential to validate and generalize these findings across imaging platforms and patient populations.

## Supplementary Information

Below is the link to the electronic supplementary material.

**Supplementary Material 1: Figure S1.** Specific growth rates (SGR) of cochlea-vestibular schwannomas in the study. In all ears with normal hearing at study initiation (*n* = 35), older patients appeared to have slower initial growth (R^2^ = 0.1219, *p* = 0.04) (**A**). However, higher SGR at study initiation did not predict eventual hearing loss (**B**). We separated those ears with sustained normal hearing (*n* = 26) and those with eventual hearing loss (*n* = 9). We found no difference between these two groups with initial SGR or age (R^2^ = 0.1083 and 0.2276, respectively)

**Supplementary Material 2: Figure S2.** Representative image set showing non-contrasted FLAIR (left column), T2-weighted (middle column), and T1 with-contrast (right column). T1 with-contrast demonstrates tumor volume, T2-weighted shows partial IAC occlusion, and non-contrasted FLAIR demonstrates hyper-intensity within the basal turn of the cochlea. Yellow-arrowheads denote the affected side. Bottom section of figure shows results of Fisher’s Exact test for IAC occlusion vs. FLAIR, demonstrating that IAC occlusion at study entry was associated with intralabyrinthine FLAIR hyper-intensity

**Supplementary Material 3: Figure S3.** Representative imaging course for NF2 subject with T1 with-contrast (top), T2-weighted (middle), and non-contrasted FLAIR images (bottom). IAC occlusion and FLAIR hyper-intensity was identified in the right ear. Audiometry results are noted showing that a mild degree of hearing loss occurred after two years of normal hearing with IAC occlusion paired with FLAIR hyper-intensity

**Supplementary Material 4: Figure S4.** Hearing outcomes for patients with positive intralabyrinthine FLAIR hyperintensity who subsequently underwent middle fossa decompression. (**A**) Rate of change in 4f-PTA scores and (**B**) word recognition scores for patients undergoing middle fossa decompression surgery (at year 0) before and after surgery

## Data Availability

The human data generated in this study are not publicly available due to patient privacy requirements but are available upon reasonable request from the corresponding author. Other data generated in this study are available within the article and its Supplemental data files.
