# Supplementary figures and images for: Intralabyrinthine MRI FLAIR as a predictive marker for hearing loss in vestibular schwannomas in Neurofibromatosis Type 2
Source: J Neurooncol. 2026 Jan 26;176(3):182. doi: 10.1007/s11060-026-05422-9 (PMC12835039; doi:10.1007/s11060-026-05422-9)

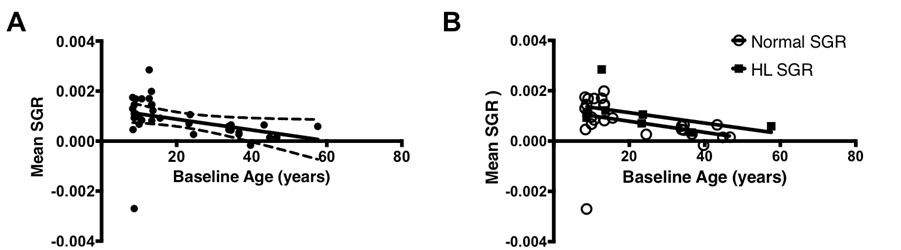

Supplement: Supplementary file 1 — Supplementary Material 1: Figure S1. Specific growth rates (SGR) of cochlea-vestibular schwannomas in the study. In all ears with normal hearing at study initiation (n = 35), older patients appeared to have slower initial growth (R2 = 0.1219, p = 0.04) (A). However, higher SGR at study initiation did not predict eventual hearing loss (B). We separated those ears with sustained normal hearing (n = 26) and those with eventual hearing loss (n = 9). We found no difference between these two groups with initial SGR or age (R2 = 0.1083 and 0.2276, respectively) [file 11060_2026_5422_MOESM1_ESM.tif]

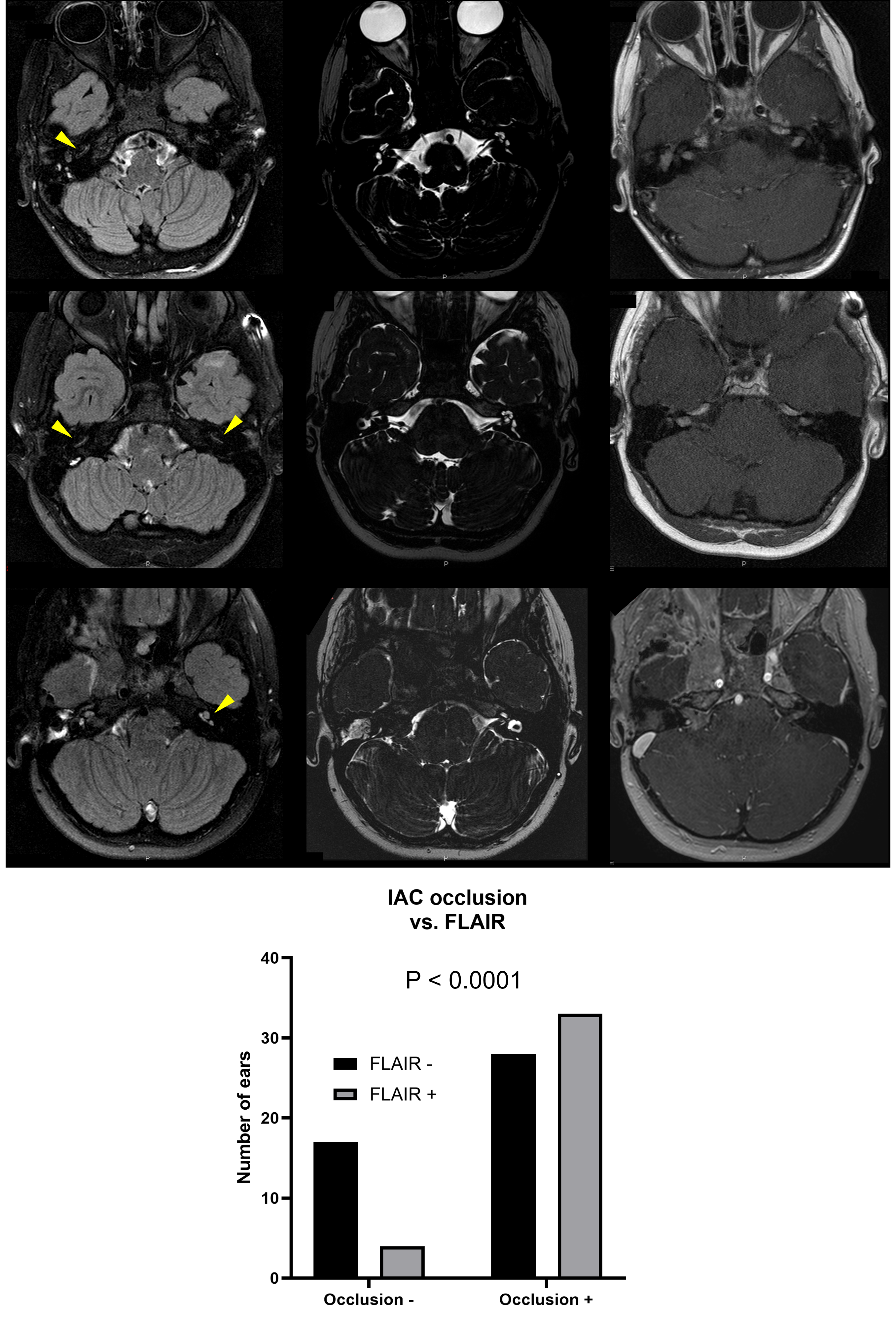

Supplement: Supplementary file 2 — Supplementary Material 2: Figure S2. Representative image set showing non-contrasted FLAIR (left column), T2-weighted (middle column), and T1 with-contrast (right column). T1 with-contrast demonstrates tumor volume, T2-weighted shows partial IAC occlusion, and non-contrasted FLAIR demonstrates hyper-intensity within the basal turn of the cochlea. Yellow-arrowheads denote the affected side. Bottom section of figure shows results of Fisher’s Exact test for IAC occlusion vs. FLAIR, demonstrating that IAC occlusion at study entry was associated with intralabyrinthine FLAIR hyper-intensity [file 11060_2026_5422_MOESM2_ESM.tif]

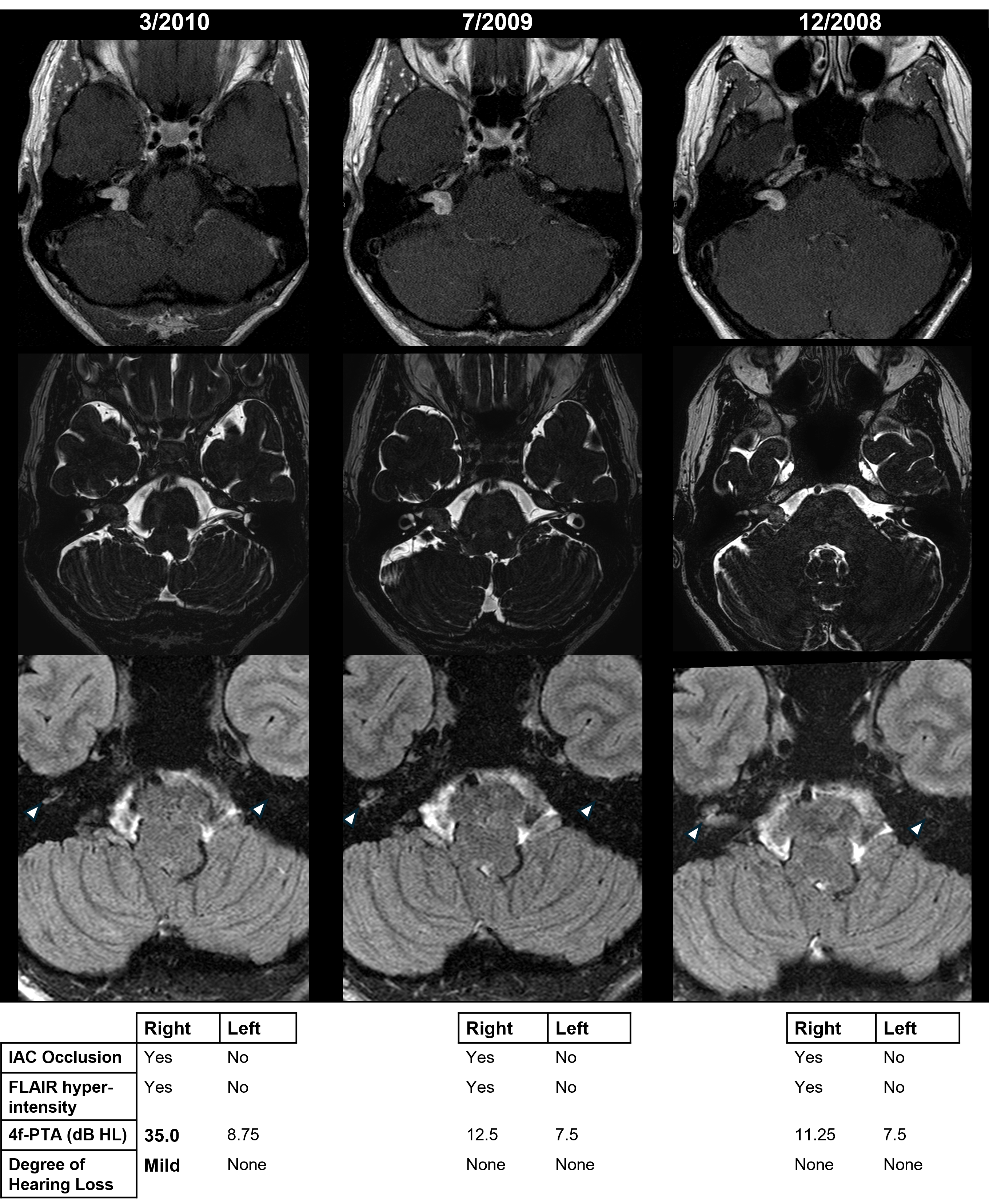

Supplement: Supplementary file 3 — Supplementary Material 3: Figure S3. Representative imaging course for NF2 subject with T1 with-contrast (top), T2-weighted (middle), and non-contrasted FLAIR images (bottom). IAC occlusion and FLAIR hyper-intensity was identified in the right ear. Audiometry results are noted showing that a mild degree of hearing loss occurred after two years of normal hearing with IAC occlusion paired with FLAIR hyper-intensity [file 11060_2026_5422_MOESM3_ESM.tif]

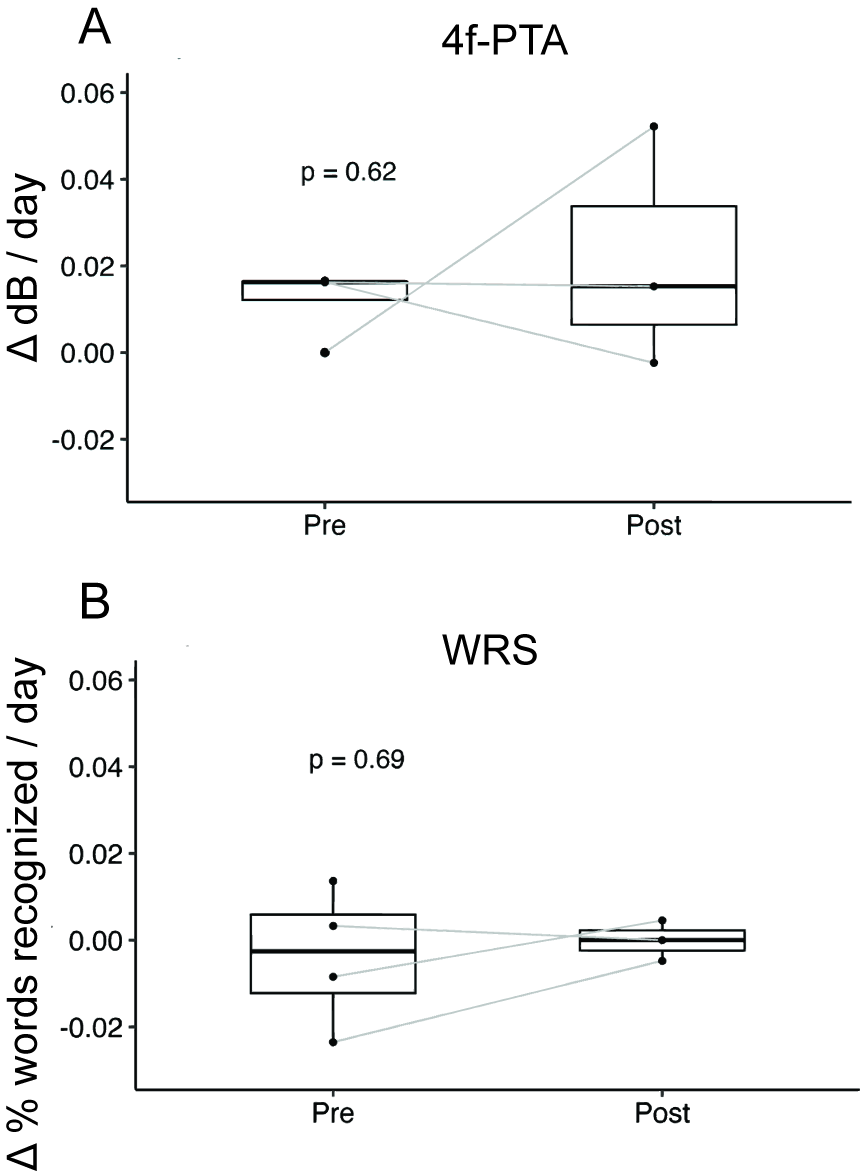

Supplement: Supplementary file 4 — Supplementary Material 4: Figure S4. Hearing outcomes for patients with positive intralabyrinthine FLAIR hyperintensity who subsequently underwent middle fossa decompression. (A) Rate of change in 4f-PTA scores and (B) word recognition scores for patients undergoing middle fossa decompression surgery (at year 0) before and after surgery [file 11060_2026_5422_MOESM4_ESM.tif]
